# Mindfulness-based stress reduction for people with multiple sclerosis – a feasibility randomised controlled trial

**DOI:** 10.1186/s12883-017-0880-8

**Published:** 2017-05-16

**Authors:** Robert Simpson, Frances S. Mair, Stewart W. Mercer

**Affiliations:** General Practice and Primary Care, Institute of Health and Wellbeing, University of Glasgow, House 1, 1 Horselethill Road, Glasgow, Scotland G12 9LX UK

## Abstract

**Background:**

Multiple sclerosis (MS) is a stressful condition. Mental health comorbidity is common. Stress can increase the risk of depression, reduce quality of life (QOL), and possibly exacerbate disease activity in MS. Mindfulness-Based Stress Reduction (MBSR) may help, but has been little studied in MS, particularly among more disabled individuals.

**Methods:**

The objective of this study was to test the feasibility and likely effectiveness of a standard MBSR course for people with MS. Participant eligibility included: age > 18, any type of MS, an Expanded Disability Status Scale (EDSS) </= 7.0. Participants received either MBSR or wait-list control. Outcome measures were collected at baseline, post-intervention, and three-months later. Primary outcomes were perceived stress and QOL. Secondary outcomes were common MS symptoms, mindfulness, and self-compassion.

**Results:**

Fifty participants were recruited and randomised (25 per group). Trial retention and outcome measure completion rates were 90% at post-intervention, and 88% at 3 months. Sixty percent of participants completed the course. Immediately post-MBSR, perceived stress improved with a large effect size (ES 0.93; *p* < 0.01), compared to very small beneficial effects on QOL (ES 0.17; *p* = 0.48). Depression (ES 1.35; *p* < 0.05), positive affect (ES 0.87; *p* = 0.13), anxiety (ES 0.85; *p* = 0.05), and self-compassion (ES 0.80; *p* < 0.01) also improved with large effect sizes. At three-months post-MBSR (study endpoint) improvements in perceived stress were diminished to a small effect size (ES 0.26; *p* = 0.39), were negligible for QOL (ES 0.08; *p* = 0.71), but were large for mindfulness (ES 1.13; *p* < 0.001), positive affect (ES 0.90; *p* = 0.54), self-compassion (ES 0.83; *p* < 0.05), anxiety (ES 0.82; *p* = 0.15), and prospective memory (ES 0.81; *p* < 0.05).

**Conclusions:**

Recruitment, retention, and data collection demonstrate that a RCT of MBSR is feasible for people with MS. Trends towards improved outcomes suggest that a larger definitive RCT may be warranted. However, optimisation changes may be required to render more stable the beneficial treatment effects on stress and depression.

**Trial registration:**

ClinicalTrials.gov Identifier NCT02136485; trial registered 1st May 2014.

**Electronic supplementary material:**

The online version of this article (doi:10.1186/s12883-017-0880-8) contains supplementary material, which is available to authorized users.

## Background

Multiple sclerosis (MS) is a stressful condition, with unpredictable relapses and disease progression [1], problematic comorbidity [2], complex drug regimens [3], and manifold social and role difficulties [1]. Stress may contribute to disease activity in MS [4, 5], is burdensome, can negate health-promoting behaviors [6], may contribute to the development of anxiety and depression and impair quality of life (QOL) in MS [7, 8]. Anxiety and depression are common in MS [2]. Evidence to support the use of pharmacological treatments for mental health comorbidities in MS is limited, with problematic side effects noted [9]. Amongst psychological interventions, cognitive behavioural therapy (CBT) approaches are often used, but evidence is limited and reviews consistently highlight a need for further high quality research to identify other effective and acceptable interventions [10]. CBT has the strongest evidence for treating depression [10] and stress [11] in MS, but effective treatments for anxiety are lacking [10].

Mindfulness-based interventions (MBIs) are increasingly used to manage mental health problems and stress in long-term conditions (LTCs), with meta-analytic studies reporting comparable efficacy to both CBT and antidepressants [12]. MBIs are multicomponent complex interventions that teach a variety of meditation skills to facilitate the development of ‘mindfulness’ [13]. The term mindfulness is often defined as; ‘*paying attention in a particular way: on purpose, in the present moment, and non-judgmentally*’ [14]. How MBIs work is not fully understood, but improved executive skills and enhanced emotion regulation are thought key mechanisms [15, 16]. Mindfulness-Based Stress Reduction (MBSR) was the original model for MBIs, first used for people with chronic pain [17]. MBIs have since been applied to a wide range of other LTCs, with existing evidence supporting their use in anxiety [18], recurrent depression [18], and somatisation [19].

In people with MS, a recent systematic review found limited evidence that MBIs may improve anxiety, depression, pain, fatigue, balance, and QOL [20]. Since then, further evidence suggests potential cost-effectiveness [21]. However, how best MBIs should be delivered to diverse MS populations remains unclear. Differing demographic factors such as age, sex, socio-economic status (SES), ethnicity, comorbidity, and greater levels of disability could conceivably impact on effectiveness. Prior studies have largely focused on distinct disease phenotypes [21, 22], or less disabled individuals [23–27], meaning that previous findings suggesting benefit from MBSR [24] may not apply to the wider spectrum of people with MS. Only one prior study has reported detailed feasibility findings for the use of MBIs in people with MS, where data is limited to people with progressive disease [21]. Very little is thus known about the feasibility, acceptability, accessibility, and implementability of standard MBSR for people with MS.

The aim of the study described in this paper was to determine the feasibility of conducting a definitive phase-3 randomised controlled trial (RCT) of standard MBSR and to obtain initial estimates of likely effectiveness.

## Methods

### Trial design and participants

This was a phase-2 exploratory RCT to assess the feasibility (engagement and retention), and likely effectiveness of MBSR in people with MS. It was conducted in Glasgow, Scotland, United Kingdom (UK). Acceptability, accessibility, and implementability were also assessed via nested qualitative semi-structured interviews (recently submitted for publication) and will be reported elsewhere.

The study employed a wait-list control design, meaning that all study participants eventually received MBSR. We aimed to recruit 50 adults with MS within 3 months. Between June and August 2014, participants were recruited from National Health Service (NHS) sites in Greater Glasgow. Participants were recruited directly by clinical staff on NHS sites providing MS services; by alerting all GPs in this health board area by email; advertising via NHS/third-sector bodies (MS Revive); through internet adverts (MS Society UK), and the University of Glasgow Twitter/Facebook social media outlets. Full inclusion and exclusion criteria are provided below (Table 1).

**Table 1:** Study eligibility criteria

Inclusion	1) Over 18 years of age; 2) Neurologist confirmed diagnosis of MS (Poser or McDonald criteria depending on year of diagnosis); 3) Able to understand spoken and written English; 4) A score of less than or equal to 7.0 on the Expanded Disability Status Scale (EDSS) [49]
Exclusion	1) Life-threatening physical or mental health comorbidities (i.e. suicidal ideation, active psychosis, or terminal/life threatening inter-current medical illness), or such conditions expected to significantly limit participation and adherence (eg dementia, pregnancy, on going substance abuse); 2) Those currently receiving another form of psychological intervention (non-pharmacological).

A priori stopping criteria were for trial discontinuation in the occurrence of any adverse event(s) strongly suggesting harm from the intervention.

Recruited participants had baseline measures and informed consent collected face-to-face, prior to randomisation. All measures were self-completed; firstly with a researcher present, then on subsequent iterations by the participant alone. Follow-up measures were collected by post, at intervention completion (2 months), and then again 3 months later (at 5 months). Those who did not return postal measures within 14 days were telephoned to confirm ongoing participation. After waiting 2 weeks for response, the average number of reminder calls required was 4.4. Participants received a £5 gift voucher as a gesture of appreciation for completing each questionnaire.

### Randomisation and blinding

An independent, blinded statistician from the Robertson Centre for Biostatistics (RCB) at the University of Glasgow undertook randomisation and sequence generation, once all baseline measures had been collected. Block sizes of two were generated, to prevent over-allocation to either group. Blinded staff at the University of Glasgow with no prior knowledge of participants handled treatment allocation. Questionnaire data was identifiable only by a random study number, and data entry for all outcome measures was done by a researcher blinded to group allocation. It was impossible to blind participants or MBSR instructors to their treatment allocation.

### Patient involvement

The trial protocol was prospectively reviewed by three patient members of the UK MS Society research network who provided favourable feedback for the study.

### Intervention

The intervention was based on standard MBSR, including home practice materials, but without the day retreat at week six; excluded for pragmatic, space-constraint reasons, as well as empirical evidence contesting its necessity [28] (Additional file 1). All MBSR classes were led by two experienced physician facilitators and took place at the NHS Centre for Integrative Care (NHS CIC), in Glasgow.

### MBSR instructors

The MBSR instructors were used to working together. Over the previous 3 years they had regularly taught weekly mindfulness groups to multimorbid people with a variety of LTCs (including MS) as part of their routine clinical responsibilities. However, neither instructor had taught groups exclusively for people with MS.

The first MBSR instructor had a clinical background in General Practice since 1983 and worked full time as a Specialty Doctor at the NHS CIC. She had completed teacher training in Mindfulness-Based Cognitive Therapy (MBCT) in 2005, via the University of Bangor, and had been teaching mindfulness regularly since then. She had also completed residential MBSR training in with Jon-Kabat-Zinn, in the USA in 2011, and regularly attended training retreats in MBCT and Vipasana meditation. She had a teaching qualification in Pranayama and Iyengar Yoga. She regularly attended one-to-one mindfulness clinical supervision and had a longstanding daily practice.

The second MBSR instructor also came from a General Practice background, completing her clinical training in 1994. She qualified as a mindfulness teacher in 2011 via the University of Bangor. She was halfway through completing an MSc in teaching mindfulness via the University of Bangor and had a longstanding daily mindfulness practice with regular clinical supervision.

### Intervention fidelity

Fidelity assessment was informed by the National Institutes of Health guidance for behavioural interventions [29] (Table 2).

**Table 2:** Treatment fidelity

Domain of fidelity	How it was met
1. Study design	A priori study protocol; fixed number/length of MBSR sessions; recording of any protocol deviations; scripted manual for course; external monitoring by research team and MBSR instructor not part of the research project; monitoring homework completion
2. Provider training	Qualified and experienced mindfulness teachers trained together using standardised MBSR treatment manuals; same instructors throughout; regular external provider debriefing and supervision; easy access to senior research staff (SM); participant exit interviews enquiring about intervention content
3. Improving delivery of MBSR	Qualitative assessment of provider ‘warmth/credibility’ from participants, complaint monitoring; treatment workbook provided to all participants;
4. Improving receipt of MBSR	Providers asked for weekly participant feedback, both verbal, and in writing (embedded questionnaire – not part of study data); completion of regular activity logs; participant and provider feedback on MBSR exercises during classes; telephone follow-up with drop-outs
5. Improving MBSR skill enactment	Semi-structured participant interviews on completion; regular home practice and materials provided along with diary for adherence; in class discussion/post-interview discussion on ongoing use/application of MBSR skills in daily life

However, the UK Medical Research Council (MRC) guidance for developing and evaluating complex interventions [13] suggests that any such measures should be flexible during the early stages of evaluation of a novel intervention/context. For example, there was no ‘reviewer’ sitting in or video-recording the classes, something that would be important in a full-scale trial.

### Outcome objectives

As a feasibility study, the main outcome objectives were:

1. To determine if recruitment, delivery, and retention for a RCT of MBSR was feasible
2. To determine if outcome measurement data collection was feasible
3. To assess likely effectiveness on outcome measures in a definitive trial

The primary participant-report outcomes were:

- Perceived stress (Perceived Stress Scale-10 – PSS [30])
- Quality of life (EQ-5D-5 L) [31].

Secondary participant-report outcomes sought to:

1. Capture common MS symptoms with a MS specific measure (Multiple Sclerosis Quality of Life Inventory - MSQLI) [32]. The MSQLI includes measures of:
  - Fatigue (cognitive, physical, psychosocial) (Modified Fatigue Impact Scale – MFIS)
  - Mental health (anxiety, depression, behavioural control, positive affect) (Mental Health Inventory-18 - MHI)
  - Social support (tangible, emotional, affection, positive interactions) (Modified Social Support Survey - MSSS)
  - Cognitive function (attention, retrospective memory, prospective memory, planning) (Perceived Deficits Questionnaire - PDQ)
  - Pain (Pain Effects Scale – PES)
  - Visual function (Impact of Visual Impairment Scale - IVIS)
  - Bladder function (Bladder Control Scale - BCS)
  - Bowel function (Bowel Control Scale - BWCS)
  - Sexual satisfaction (Sexual Satisfaction Scale – SSS)

2. Assess items measuring the putative processes of mindfulness:
  - Mindfulness (Mindful Attention Awareness Scale – MAAS) [33].
  - Self-compassion (Self-Compassion Scale - short form – SCS-sf) [34].

3. Measure emotional lability via the Emotional Lability Questionnaire (ELQ) [35].

For psychometric properties and justification of measures used, see Additional file 2.

A baseline participant questionnaire also recorded demographic information, including deprivation, as measured by the Scottish Index of Multiple Deprivation (SIMD) [36], number of comorbid conditions, and use of disease-modifying, antidepressant, or analgesic medications.

### Statistical analysis

An a priori statistical analysis plan was developed in conjunction with a Consultant Biostatistician from the Robertson Centre for Biostatistics, University of Glasgow. This was made publicly available online, in advance of all data collection: http://www.gla.ac.uk/researchinstitutes/healthwellbeing/research/generalpractice/research/mbsr-ms/.

Key outcomes to assess feasibility, acceptability, and accessibility included rates of recruitment and retention. These were recorded using descriptive statistics.

Baseline characteristics were summarised by intervention arm.

Differences were tested via two-sample t-tests for normally distributed variables, and chi-squared tests for categorical variables. Questionnaire outcome data were analysed relative to change from baseline via an analysis of covariance (ANCOVA) approach, where adjustments were made for the baseline score, as appropriate, including age, sex, and deprivation, as well as any other characteristics found to differ between the intervention arms at baseline. On the advice of the statistician, age, sex, and SES were included as common confounders, however, unadjusted analyses were also undertaken for comparison.

An a priori decision was made not to perform data imputation for missing values. There were no interim analyses performed. All statistical analyses were undertaken using SPSS v21.

For questionnaire data, results are reported for between group mean (standard deviation - SD) baseline scores, change scores, treatment effects (β) with 95% confidence intervals (95% CIs), significance levels, and effect sizes (ES) (Cohen’s ‘d’) with 95% CIs.

### Sample size calculation

Given the aim of this study being to assess feasibility and power estimate for a phase-3 trial, sample size was not based on a power calculation. However, based on the advice of a statistician, a working sample size of 50 people was chosen as:

1. Browne [37] has demonstrated that an ‘n’ of 30 is sufficient to allow estimates of sample size for an efficacy trial.
2. Pragmatic reasons including a) the MBSR instructors were used to routinely delivering groups of this size; and b) space constraints meant that a maximum of 25 participants could be accommodated in each group.

### Data access

Data for the trial was accessible to all authors (RS, FM, SM), who each contributed to its interpretation.

## Results

### Objective 1 - To determine if recruitment, delivery, and retention for a large scale RCT of MBSR were feasible

In total, 101 patients were approached, of whom 66 (65%) contacted the researcher (RS) and were screened. Following screening, three people were ineligible (EDSS > 7.0), and 13 declined to take part due to: difficulties securing transport (*n* = 7); difficulty getting time off work (*n* = 4); thought the course would be too tiring (*n* = 1); unclear (*n* = 1). Consent rate was thus 50/66, or 76%. Recruitment was completed within 10 weeks (out of the pre-specified 12) (Table 3).

**Table 3:** Sources of trial recruitment and relative contributions

Source of engagement/ recruitment	Numbers known to have been approached	Numbers (known) expressing interest	Numbers recruited into trial (n/50)	Percentage of recruitment overall
MS Specialist Nurses	75	52	34	68%
MS Revive Nurse	6	6	6	12%
Integrative Medicine Specialists	9	9	5	10%
General practitioners	11	11	5	10%
Via MS Society advertisement	Freely available online	2	0	0%
Via University web (Twitter/ Facebook)	Freely available online	0	0	0%
Via protocol (clinical trials.gov)	Freely available online	5	0	0%
Total	101(+)	85	50	N/A

**Fig. 1:**
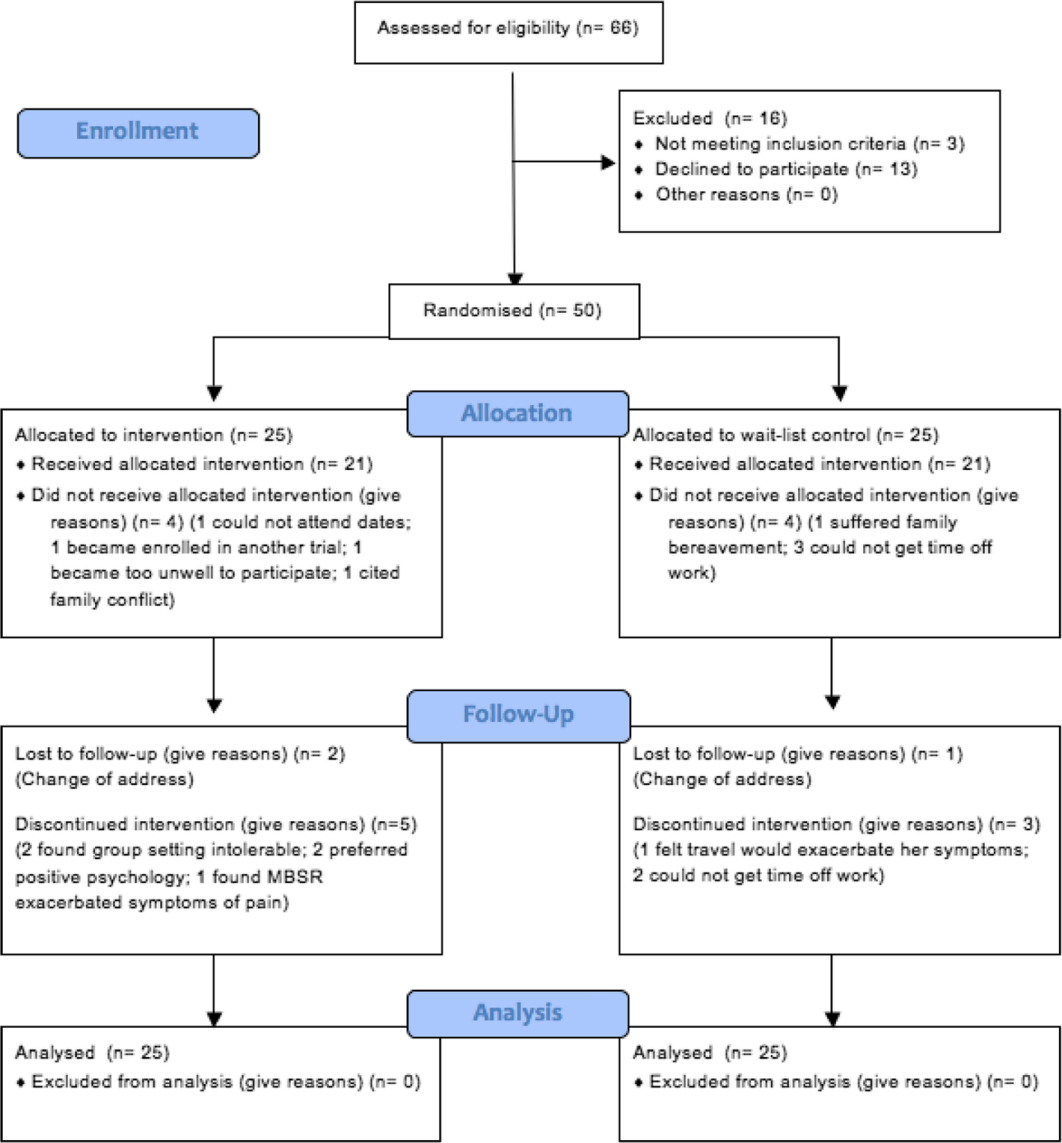
CONSORT flow diagram

Following randomisation, one participant assigned to MBSR could not attend the allocated dates, and thus did not receive the intervention, but continued to complete measures throughout, and another three participants withdrew due to: a) a MS symptom exacerbation (fatigue), b) family conflict, and c) becoming enrolled in a pharmacological trial. Thus, from the 25 participants originally assigned to the intervention, only 21 could attend any classes. See Fig. 1 below for a detailed CONSORT flow diagram.

### Treatment adherence

Treatment adherence was assessed via attendance at the MBSR sessions, and via home practice recordings. Fifteen participants (60%) attended four or more MBSR sessions, meeting the criteria for course ‘completion’. There were no significant differences in demographic factors between completers and non-completers (Additional file 3). Reasons cited for session non-attendance included inter-current illness, work commitments, being on holiday, and ‘sleeping-in’. See Table 4 for details of MBSR session attendance.

**Table 4:** MBSR session attendance

MBSR sessions completed	Number of participants	Percentage (%)
All	3	12%
7	8	32%
6	3	12%
5	1	4%
4	0	0%
3	1	4%
2	1	4%
1	4	16%
0	4	16%

Participants were asked to return a home-practice log each week, but only 16/25 (60%) people returned any data. From these, an average home practice time of 32.5 min per day was observed. Four people allocated to MBSR did not attend any sessions, and provided no data on home-practice.

### Baseline participant characteristics

The sample at baseline had a mean age (SD) of 44.96 (10.90), was predominantly female (92%), and of White Scottish ethnicity (98%). Forty participants (80%) had Relapsing Remitting MS (RRMS), eight (16%) had Secondary Progressive (SPMS), and two (4%) had Primary Progressive disease (PPMS). The mean (SD) EDDS was 4.41 (1.75). After randomisation, the intervention and control groups were similar in terms of age, sex, SES, ethnicity, level of education, MS phenotype, disease duration, number of comorbidities, and EDSS score, with the only significant baseline difference relating to previous meditation/yoga experience, which by chance was higher in the intervention group. Thus, this potential confounder was controlled for in the subsequent analyses, along with age, sex, and SES. Baseline demographic and clinical data are summarised in Table 5.

**Table 5:** Baseline participant characteristics

	Intervention	Control	Significance p
Mean age in years (standard deviation - SD)	43.6 (10.7)	46.3 (11.1)	0.37
Sex	Female 23 (92%)	Female 22 (88%)	1.00
Ethnicity	White British 25 (100%)	White British 25 (100%)	1.00
MS phenotype RRMS – relapsing remitting; SPMS – secondary progressive; PPMS – primary progressive	RRMS 22 (88%)	RRMS 18 (72%)	0.74
	SPMS 1 (4%)	SPMS 7 (28%)	
	PPMS 2 (8%)		
Deprivation	5.0 (2.8)	5.4 (2.6)	0.64
Education – highest level	Secondary school 3 (12%)	Secondary school 5 (20%)	0.73
	College 7 (28%)	College 7 (28%)	
	University 15 (60%)	University 13 (52%)	
Employment	Full time 4 (16%)	Full time 7 (28%)	0.39
	Part time 3 (12%)	Part time 6 (24%)	
	Unemployed 6 (24%)	Unemployed 7 (28%)	
	Retired 5 (20%)	Retired 3 (12%)	
	Other 7 (28%)	Other 2 (8%)	
Living arrangement	Lives alone 6 (24%)	Lives alone 3 (12%)	0.54
	With partner 9 (36%)	With partner 10 (40%)	
	With family/friends 10 (40%)	With family/friends 12 (48%)	
EDSS	4.5 (1.8)	4.3 (1.7)	0.64
Mean disease duration in years (SD)	8.9 (8.5)	9.6 (9.4)	0.79
Mean total comorbidity count (SD)	2.5 (2.2)	2.3 (1.9)	0.68
Mean mental health comorbidity count (SD)	0.8 (0.83)	0.7 (0.8)	0.73
• Comorbid anxiety	11 (44%)	8 (32%)	0.12
• Comorbid depression	9 (36%)	11 (44%)	0.29
Mean physical health comorbidity count (SD)	1.8 (1.5)	1.6 (1.5)	0.71
Using analgesic drugs	19 (76%)	17 (68%)	0.75
Using disease modifying drugs	14 (56%)	12 (48%)	0.78
Using antidepressant drugs	12 (48%)	11 (44%)	1.00
Previous meditation/yoga experience	17 (68%)	10 (40%)	*0.04*

*Statistically significant difference

### Objective 2 - To determine if outcome measurement data collection was feasible

All 50 participants completed outcome measures at baseline (100%). At the post intervention point (two-months) 45/50 (90%) returned these measures, and at the final follow-up point (five-months) 44/50 (88%) were returned. Over the entire period for collecting post-intervention and follow-up measures, an average of 4.4 telephone reminders were required per participant (222 calls in total). Missing data varied considerably across the range of outcome measures. Thirty-nine out of 50 (78%) participants returned at least one item of missing data, and it was commoner among measures towards the back of the questionnaire. It was lowest on the EQ-5D-5 L (0–12%), and highest for the SCS-sf (2–22%), with 13/15 measures having less than 20% missing values. There were no significant differences in age (Mean age 47.12 [SD 10.44] vs. 44.96 [SD 10.90]; *p* = 0.39), disability level (Mean EDSS 4.51 [SD 1.77] vs. 4.41 [SD 1.75]; *p* = 0.40), or SES (Mean deprivation decile 5.08 [SD 2.64] vs. 5.22 [SD 2.71]; *p* = 0.60) between those returning missing values, and those who did not. See Additional file 4 for further details regarding missing data.

### Objective 3 - To assess likely effectiveness on outcome measures in a definitive trial

There were no significant differences between the groups on any of the baseline outcome measures. Two models were explored in the analyses 1) a ‘raw’ model that made no adjustments for demographic factors; and 2) a model that adjusted for age, sex, SES, and previous yoga/meditation experience. There were only minor differences between the models. In the raw model beneficial effect sizes were slightly larger on all measures (Additional file 5). As such, the model presented below is that adjusted for age, sex, deprivation and previous yoga/meditation experience.

**Fig. 2:**
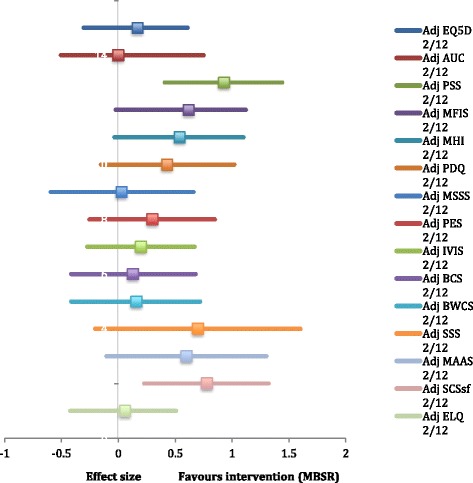
Effect sizes with 95% confidence intervals immediately post-MBSR (adjusted for age/sex/SES/previous meditation/yoga): EQ5D – EuroQol QOL measure adjusted for age/sex/SES/meditation/yoga. AUC – EuroQol area under the curve adjusted for age/sex/SES/meditation/yoga. PSS – Perceived stress scale adjusted for age/sex/SES/meditation/yoga. MFIS – Modified fatigue impact scale adjusted for age/sex/SES/meditation/yoga. MHI – Mental health inventory adjusted for age/sex/SES/meditation/yoga. PDQ – Perceived deficits questionnaire adjusted for age/sex/SES/meditation/yoga. MSSS – Modified social support survey adjusted for age/sex/SES/meditation/yoga. PES – Pain effects scale adjusted for age/sex/SES/meditation/yoga. IVIS – Impact of visual impairment scale adjusted for age/sex/SES/meditation/yoga. BCS – Bladder control scale adjusted for age/sex/SES/meditation/yoga. BWCS – Bowel control scale adjusted for age/sex/SES/meditation/yoga. SSS – Sexual satisfaction scale adjusted for age/sex/SES/meditation/yoga. MAAS – Mindful attention awareness scale adjusted for age/sex/SES/meditation/yoga. SCS-sf – Self-compassion scale – short form adjusted for age/sex/SES/meditation/yoga. ELQ – Emotional lability questionnaire adjusted for age/sex/SES/meditation/yoga

In the model adjusted for age, sex, SES, and previous meditation/yoga experience, at immediately post-MBSR, PSS scores improved with a large effect size (ES 0.93; *p* < 0.05), but EQ-5D-5 L scores showed only very small improvement (ES 0.17; *p* = 0.48). From the secondary outcomes, improvements with a large effect size were evident for depression (ES 1.35; *p* < 0.05), positive affect (ES 0.87; *p* = 0.13), anxiety (ES 0.85; *p* = 0.05), and self-compassion (ES 0.80; *p* < 0.01). Overall, 14 out of 15 of the composite outcome measures (primary and secondary) showed a positive trend for treatment effect immediately post-MBSR (Fig. 2).

**Fig. 3:**
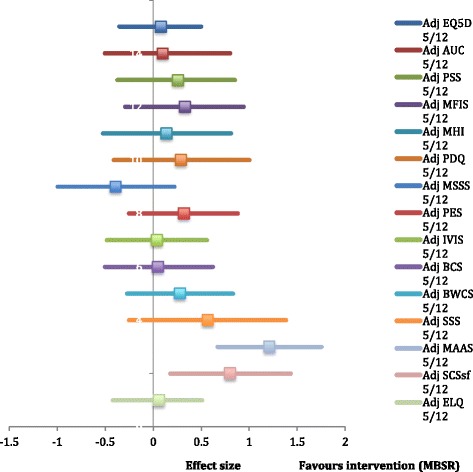
Effect sizes with 95% confidence intervals 3 months post-MBSR (adjusted for age/sex/SES/previous meditation/yoga): EQ5D – EuroQol QOL measure adjusted for age/sex/SES/meditation/yoga. AUC – EuroQol area under the curve adjusted for age/sex/SES/meditation/yoga. PSS – Perceived stress scale adjusted for age/sex/SES/meditation/yoga. MFIS – Modified fatigue impact scale adjusted for age/sex/SES/meditation/yoga. MHI – Mental health inventory adjusted for age/sex/SES/meditation/yoga. PDQ – Perceived deficits questionnaire adjusted for age/sex/SES/meditation/yoga. MSSS – Modified social support survey adjusted for age/sex/SES/meditation/yoga. PES – Pain effects scale adjusted for age/sex/SES/meditation/yoga. IVIS – Impact of visual impairment scale adjusted for age/sex/SES/meditation/yoga. BCS – Bladder control scale adjusted for age/sex/SES/meditation/yoga. BWCS – Bowel control scale adjusted for age/sex/SES/meditation/yoga. SSS – Sexual satisfaction scale adjusted for age/sex/SES/meditation/yoga. MAAS – Mindful attention awareness scale adjusted for age/sex/SES/meditation/yoga. SCS-sf – Self-compassion scale – short form adjusted for age/sex/SES/meditation/yoga. ELQ – Emotional lability questionnaire adjusted for age/sex/SES/meditation/yoga

At follow-up, 3 months following MBSR, beneficial effects on the PSS had diminished to a small effect size (ES 0.26; *p* = 0.39) and those for the EQ-5D-5 L were negligible (0.08; *p* = 0.71). From secondary outcomes, improvements with a large effect size were evident for mindfulness (ES 1.21; *p* < 0.001), positive affect (ES 0.90; *p* = 0.54), anxiety (ES 0.82; *p* = 0.15), prospective memory (ES 0.81; *p* < 0.05), and self-compassion (ES 0.80; *p* < 0.05). There was an overall trend towards improvement on 14 out of 15 of the composite measures (Fig. 3).

See Figs. 2 and 3 for a graphical summary of treatment effects for adjusted models covering the PSS, EQ-5D-5 L, the MSQLI, MAAS, SCS-sf, and the ELQ. Further statistical details are outlined in Tables AF6.1–AF6.9 in Additional file 6.

### Harms

One participant allocated to the intervention group reported an exacerbation in her symptoms of neuropathic pain following her first and only MBSR class.

## Discussion

This study assessed the feasibility of delivering MBSR for people with MS in an exploratory phase-2 RCT. We recruited our target of 50 participants within 3 months. Very high rates of study retention were achieved, with 45 (90%) participants completing outcome measures immediately post-intervention, and 44 (88%) at follow-up, 3 months later. Missing values were generally low. MBSR session attendance was only 60%, less than that reported in other studies of MBIs for people with MS (range 92–95%) [21, 24].

Regarding primary efficacy outcomes, large initial improvements were seen in perceived stress at post-intervention in this study, but diminished to small at three-month follow-up. Improvements in QOL were very small at post-intervention and negligible at three-month follow-up. For secondary outcomes, large improvements in depression, positive affect, anxiety, and self-compassion were apparent immediately post-MBSR, whilst at three-month follow-up large improvements were present for mindfulness, positive affect, anxiety, prospective memory, and self-compassion.

### Comparison with existing literature

A large RCT testing MBSR in less disabled patients with MS that took place in a university hospital location reported a lower refusal rate (9%) than the current study (24%), but they used an advertisement-based ‘opt-in’ approach to recruitment, whereas the current study employed a multifaceted strategy with a greater potential to record refusal [24]. Outcome measure completion in their study was 91% at six-month follow-up, versus 88% in ours at 3 months post MBSR. They also reported much higher session attendance (92%) than the current study (60%), perhaps reflecting lower levels of disability in their participants (mean EDSS 3.0; SD 1.0 versus mean EDSS 4.41; SD 1.75), or slightly higher level of mindfulness teacher experience (>9 years versus 7.5). They delivered MBSR in smaller groups (*n* = 10–15 per course), versus the relatively large group size in this current study (*n* = 25). Average home-practice times in their study (29.2 min) were comparable to those in this current study (32.5 min).

No previous research studies have demonstrated improvements in cognitive function among MS patients following mindfulness training. With respect to mental health outcomes, a high quality powered RCT tested MBSR in people with MS, but did not control for potential confounders in their analyses [24]. The authors similarly reported improvements in mental health, with smaller beneficial effects on depression post-intervention (ES 0.65; *p* < 0.001), which diminished less than ours at six-month follow-up (ES 0.36; *p* < 0.05). They also found smaller improvements than us in anxiety post-intervention (ES 0.39; *p* < 0.001) and at six-month follow up (ES 0.36; *p* < 0.05). The authors also reported that beneficial effects on both anxiety and depression were greater when subgroup analyses focused only on those with pre-intervention scores deemed to be of clinical significance [24].

More recently, an unpowered pilot study testing a tailored remote Skype-delivered MBCT course minus mindful-movement in people with progressive MS demonstrated preliminary evidence to suggest likely effectiveness at improving mental health. Bogosian et al. [21] included only those with levels of distress on the General Health Questionnaire (GHQ) indicative of psychiatric ‘case-ness’. Improvements with a large effect size were noted in distress 3 months post MBCT (ES 0.97; *p* < 0.05); but were smaller for anxiety post intervention (ES 0.40; *p* = 0.10), increasing to large 3 months later (ES 0.86; *p* < 0.05). Improvements in depression were smaller post-intervention (ES 0.65; *p* < 0.05), but sustained 3 months later (ES 0.53; *p* < 0.05); whilst improvements in MS symptom psychological impact post-intervention were large (ES 0.99; *p* < 0.001) and sustained 3 months later (ES 1.12; *p* < 0.01).

Four other small studies have reported similar benefits in anxiety [25, 26], depression [23, 25, 26], pain [27], balance [23, 26], and QOL [23], but methodological limitations such as small sample size [26], failure to randomise [23, 27], failure to control for researcher bias [23], and selective outcome reporting [26] limit their relevance. Furthermore, modifications to their intervention approaches, such as substituting Tai Chi in place of Hatha Yoga [23, 26, 27], or not taking place in a group format [26] raise questions regarding the relevance of their findings to the current study. Nevertheless, taking the findings from the current study into account, there is now a growing body of evidence to suggest likely effectiveness of MBIs for treating anxiety in MS. The interim benefits reported on depression following MBSR in this study and one other high quality RCT [24] are also encouraging, but the decline in effect seen at study endpoints suggest that there may be scope for judicious tailoring of MBSR to render beneficial treatment effects more stable. One such measure that has worked in MBCT for depression is the provision of post-completion ‘drop-in’ sessions, where beneficial effects have persisted for up to 2 years [38].

We also measured mindfulness and self-compassion, thought key components in how MBIs work [15, 16, 22]. No previous studies in people with MS have assessed the impact of MBI training on levels of mindfulness. Meta-analysis suggests that improvements in mindfulness may mediate improved mental health in non-MS populations [39]. Cross-sectional analyses [40–42] (*n* = 69–119) support that increased mindfulness is associated with greater positive affect, improved relationship satisfaction, less anxiety, less stress, improved wellbeing and QOL, and enhanced coping in people with MS. Having more self-compassion is associated with a greater resilience to anxiety, depression and stress in other LTCs [43], and may also make a small contribution to better mental health in people with MS [22].

### Strengths and limitations

We followed the MRC guidelines for developing and evaluating complex interventions [13]. The small size of the study is a limitation, but is acceptable in feasibility work. The high proportion of female patients in this study (92%) is notable when compared with previous research in this area (range 69–100% [23–27]). This could reflect the higher incidence of stress-related mental health comorbidities in women with MS in Scotland [44], a greater stigma among men towards reporting mental health concerns [45], or that men may be less willing to participate in research studies [46].

We adjusted for common confounding variables in this study, along with those found to differ significantly at baseline. As this study was not powered to detect significant baseline differences, the findings from our analyses must thus be treated with caution (for example other variables such as baseline between group differences in diagnoses of anxiety and depression, antidepressant usage, disease activity and/or disease modifying drug (DMD) therapy could conceivably also impact on stress levels and effectiveness and should thus be considered in a future definitive study, powered to detect significant differences on these variables).

Evidence of effectiveness cannot be reliably determined in a small, unpowered sample. In this scenario, significance levels are unreliable, and observed beneficial treatment effects on stress, mood, and cognition can, at best, only be seen as an indication of likely effectiveness in a future phase three trial. Further, the novel findings of subjective improvements in cognitive function could be better contextualised if combined with a more objective neuropsychological assessment. Criticisms of commonly used objective measures of cognitive function are that they are either too short, too long, too generic, fail to detect deficits, or require to be administered by an expert [47]. The Brief International Cognitive Assessment for Multiple Sclerosis (BICAMS) is an internationally validated, standardised assessment tool with good reliability and validity, with the added advantage over other neuropsychological tests that it can be administered by a non-expert and could thus be used in a definitive phase three study [48].

The follow-up period post MBSR was relatively short in this study i.e. 3 months, and a future phase three trial could assess whether MBIs might have more lasting effects. Further optimisation work will help with the creation of a bespoke MBI for people with MS, which may improve recruitment and render beneficial treatment effects more stable.

## Conclusion

Delivering MBSR to people with MS in a NHS setting is feasible. We achieved our recruitment target of 50 people within the specified time limits. Levels of retention i.e. completion rates for outcome measures were good post-intervention, and at follow-up. Encouraging improvements were noted on various outcome measures, in particular anxiety, with most measures having acceptable levels of missing values. However, initial beneficial effects on stress and depression were greatly diminished at study endpoint and only 60% of participants completed MBSR, suggesting that further optimisation is required before proceeding to a phase-3 definitive trial.

## Additional files

Additional file 1: – MBSR; a week-by-week class overview. **Table S1.** describes the week-by-week session content for the MBSR classes in this study. (DOCX 15 kb)

Additional file 2: – Outcome measures tested for feasibility. **Table S2.** provides a list of the outcome measures used in the study, the justification for their use, and basic psychometric properties. (DOCX 16 kb)

Additional file 3: – Baseline characteristics: completers versus non-completers. **Table S3** provides an overview of participant demographics comparing MBSR course completers with non-completers. (DOCX 15 kb)

Additional file 4: – True rates for missing values for participant questionnaires. **Table S4:** provides an overview of missing data for each of the participant report outcome measures. (DOCX 15 kb)

Additional file 5: – Unadjusted RCT patient report outcome models**. Tables S5.1–S5.5.** provide detailed statistical data for unadjusted analyses. **Figure S5.1** provides a forest plot for unadjusted treatment effects immediately post-MBSR, whilst **Figure S5.2.** provides a forest plot of unadjusted treatment effects three months post-MBSR. (DOCX 30 kb)

Additional file 6: - Adjusted RCT patient report outcome models. **Tables S6.1–S6.9.** provide detailed statistical data for adjusted analyses (age, sex, SES, previous meditation/yoga experience). (DOCX 41 kb)
