# Supplementary material for: Mindfulness-based stress reduction for people with multiple sclerosis – a feasibility randomised controlled trial
Source: BMC Neurol. 2017 May 16;17:94. doi: 10.1186/s12883-017-0880-8 (PMC5434553; doi:10.1186/s12883-017-0880-8)
Supplement: Supplementary file 1 — – MBSR; a week-by-week class overview. Table S1. describes the week-by-week session content for the MBSR classes in this study. (DOCX 15 kb) [file 12883_2017_880_MOESM1_ESM.docx]

**Table S1: MBSR; a week-by-week class overview**

| **Class (week) number** | **Main themes** | **Home practice (45 minutes daily)** |
| --- | --- | --- |
| **1** | Establishment of learning contract  Theory underlying mind-body medicine and the development of self-regulatory skills  Defining mindfulness  Introduction to mindful eating (raisin exercise), mindful breathing, and mindful body awareness | Mindful body awareness via the body scan CD  Eat one meal mindfully |
| **2** | Focused dialogue, reflection on how individual perception and conditioning affects us  Affirming self-responsibility and the role of health-enhancing behaviours | Body scan CD  Short sitting practices 10-15 minutes daily  Reflecting on the integration of mindfulness into everyday life  Pleasant events diary |
| **3** | Introducing mindful movement (flexible application of core Hatha yoga postures, mindful locomotion)  Group enquiry/discussion of their experiences  Review pleasant events calendar | Alternate body scan with mindful movement CD/hand-outs  Sitting meditation practice 10-15 minutes daily  Unpleasant events diary |
| **4** | Body scan, mindful movement, sitting meditation, with emphasis on developing concentration in the practices and an enhanced field of awareness | Alternate body scan with mindful movement CD/hand-outs  Sitting meditation practice 20 minutes daily  Practicing awareness of stress reactions and behaviours, without taking action to change these  Remaining present with ‘feeling stuck/ blocking/ numbing/ shutting off’ |
| **5** | Halfway point.  Emphasis on development of adaptation skills, including problem-, emotion-, and solution-focussed approaches to coping, and how this relates to being aware, ‘staying present’, choosing more effective responses, and the development of resilience/ enhanced recovery from stressful experiences  Investigating the ‘shadow side’ of stress responses/ trying to escape difficulty | 45 minute sitting practice CD  Alternate body scan/ mindful movement  Complete difficult communications diary  Integrate mindful awareness into daily life experiences |
| **6** | Discussion around transformation of stress coping strategies, attitudes and behaviours – developing resilience  Emphasis on broadening inner resources and health-promoting attitudes and behaviours, with discussion focused on practical application  Particular emphasis on stress and knowing one’s feelings during communications, with overview of different communication styles | Alternate sitting meditation with body scan/ mindful movement CDs |
| **7** | Mountain meditation, introducing metaphors to reconceptualise the self as ‘stable’, ‘flexible’, and ‘strong’  Review of core MBSR concepts and introducing idea of choice in personal practices, but importance of maintaining 45 minutes daily  Loving kindness/ compassion meditation, with strong emphasis on silence, and developing an attitude of loving kindness towards self/others | Choice between all practice introduced thus far  Continued ‘informal’ practice in daily experiences |
| **8** | Review of core MBSR concepts.  Opportunity to ask any remaining questions.  Mostly silent sitting meditation  Guided reflection on experience of the course  Standard course evaluation forms | Optional, but strongly encouraged to continue with a daily practice of any of the core techniques learned.  Integration of practices into daily life. |
