# Supplementary material for: Mindfulness-based stress reduction for people with multiple sclerosis – a feasibility randomised controlled trial
Source: BMC Neurol. 2017 May 16;17:94. doi: 10.1186/s12883-017-0880-8 (PMC5434553; doi:10.1186/s12883-017-0880-8)
Supplement: Supplementary file 2 — – Outcome measures tested for feasibility. Table S2. provides a list of the outcome measures used in the study, the justification for their use, and basic psychometric properties. (DOCX 16 kb) [file 12883_2017_880_MOESM2_ESM.docx]

**Table S2: Outcome measures tested for feasibility**

| **Outcome measure** | **Justification for use and basic psychometric properties** |
| --- | --- |
| **Perceived stress scale (PSS)** | A generic, self-report measure of stress with good general psychometric properties (Cronbach-α reliability co-efficient 0.89; supportive convergent and divergent reliability) [53]. Has been used previously in trials of meditation in people with MS [36]. PSS-10 has the best psychometric properties in MS [31]. |
| **EQ5D5L** | A generic measure of QOL (relating to mobility, self-care, usual activities, pain, and mental health) with good general psychometric properties, and good test-re-test reliability in UK populations [54]. Has ben used previously in trials of meditation with people with MS [21] |
| **Multiple sclerosis quality of life inventory (MSQLI)** | Has not been widely used in UK populations, but performs well in North American groups [55, 56], with adequate construct validity, internal consistency, and reliability (with only the measure of visual impairment being less reliable in those with cognitive impairment). |
| **Mindful attention awareness scale (MAAS)** | The MAAS is a generic measure of mindfulness, presented as an integrated construct. It has good internal consistency (Chronbach-α 0.78-0.92), construct validity, and test-re-test reliability. |
| **Self-compassion scale-short form (SCS-sf)** | The SCS-sf has adequate internal consistency and reliability (Chronbach-α 0.86), but has never before been used in MS populations. |
| **Emotional lability scale (ELQ)** | The ELQ is a measure of emotional lability, validated for use in people with another chronic neurodegenerative condition, motor neurone disease – MND, where it performs well in European samples [57], with good internal validity. It has never been used in people with MS. |
