# Supplementary material for: Mindfulness-based stress reduction for people with multiple sclerosis – a feasibility randomised controlled trial
Source: BMC Neurol. 2017 May 16;17:94. doi: 10.1186/s12883-017-0880-8 (PMC5434553; doi:10.1186/s12883-017-0880-8)
Supplement: Supplementary file 3 — – Baseline characteristics: completers versus non-completers. Table S3 provides an overview of participant demographics comparing MBSR course completers with non-completers. (DOCX 15 kb) [file 12883_2017_880_MOESM3_ESM.docx]

**Table S3 - Baseline characteristics: completers versus non-completers**

|  | **Completers** | **Non-completers** | **Significance p** |
| --- | --- | --- | --- |
| **Mean age in years (standard deviation - SD)** | 42.47 (10.93) | 45.20 (10.77) | 0.54 |
| **Sex** | Male 2 (13%)  Female 13 (87%) | Male 1 (10%)  Female 9 (90%) | 0.80 |
| **Ethnicity** | White British 15 (100%) | White British 15 (100%) | 1.00 |
| **MS phenotype**  RRMS – relapsing remitting  SPMS – secondary progressive  PPMS – primary progressive | RRMS 14 (93%)  SPMS 0 (0%)  PPMS 1 (7%) | RRMS 8 (80%)  SPMS 1 (10%)  PPMS 1 (10%) | 0.60 |
| **Deprivation** | 5.73 (2.89) | 4.00 (2.45) | 0.13 |
| **Education – highest level** | Secondary school 2 (13%)  College 2 (13%)  University 11 (73%) | Secondary school 1 (10%)  College 5 (50%)  University 4 (40%) | 0.13 |
| **Employment** | Full time 3 (20%)  Part time 2 (13%)  Unemployed 1 (7%)  Retired 6 (40%)  Other 3 (20%) | Full time 1 (10%)  Part time 1 (10%)  Unemployed 0 (0%)  Retired 4 (40%)  Other 4 (40%) | 0.85 |
| **Living arrangement** | Lives alone 3 (20%)  With partner 6 (40%)  With family/friends 6 (40%) | Lives alone 3 (30%)  With partner (30%)  With family/friends 4 (40%) | 0.81 |
| **EDSS** | 4.53 (1.64) | 4.55 (2.10) | 0.98 |
| **Mean disease duration in years (SD)** | 11.50 (9.28) | 7.13 (7.85) | 0.22 |
| **Mean total comorbidity count (SD)** | 2.73 (2.40) | 2.20 (1.87) | 0.56 |
| **Mean mental health comorbidity count (SD)** | 0.87 (0.83) | 0.60 (0.84) | 0.44 |
| **Mean physical health comorbidity count (SD)** | 1.87 (1.68) | 1.60 (1.35) | 0.68 |
| **Using analgesic drugs** | 12 (80%) | 7 (70%) | 0.57 |
| **Using disease modifying drugs** | 9 (60%) | 5 (50%) | 0.62 |
| **Using psychotropic drugs** | 7 (47%) | 5 (50%) | 0.87 |
| **Previous meditation/yoga experience** | 10 (67%) | 7 (70%) | 0.86 |
