# Supplementary material for: Mindfulness-based stress reduction for people with multiple sclerosis – a feasibility randomised controlled trial
Source: BMC Neurol. 2017 May 16;17:94. doi: 10.1186/s12883-017-0880-8 (PMC5434553; doi:10.1186/s12883-017-0880-8)
Supplement: Supplementary file 4 — – True rates for missing values for participant questionnaires. Table S4: provides an overview of missing data for each of the participant report outcome measures. (DOCX 15 kb) [file 12883_2017_880_MOESM4_ESM.docx]

**Table S4 - True rates for missing values for participant questionnaires**.

|  | **Participant questionnaire missing values n (%)** | | |
| --- | --- | --- | --- |
| **Measure** (total number of scale items x 50) | **Baseline** | **2-months** | **5-months** |
| **EQ-5D-5L** (900) | 0 (0%) | 36 (12%) | 36 (12%) |
| **PSS-10** (1500) | 1 (0.2%) | 72 (14.4%) | 67 (13.4%) |
| **MFIS** (3150) | 3 (0.3%) | 109 (10.4%) | 126 (12%) |
| **MHI** (2700) | 3 (0.3%) | 91 (10.1%) | 109 (12.1%) |
| **PDQ** (3000) | 4 (0.4%) | 107 (10.7%) | 130 (13%) |
| **MSSS** (2700) | 3 (0.3%) | 94 (10.4%) | 110 (12.2%) |
| **PES** (900) | 3 (1%) | 31 (10.3%) | 36 (12%) |
| **IVIS** (750) | 5 (2%) | 30 (12%) | 30 (12%) |
| **BCS** (600) | 5 (2.5%) | 21 (10.5%) | 24 (12%) |
| **BWCS** (750) | 5 (2%) | 31 (12.4%) | 31 (12.4%) |
| **SSS** (750) | 24 (9.5%) | 43 (17.2%) | 42 (16.8%) |
| **MAAS** (2250) | 19 (2.5%) | 94 (12.5%) | 155 (20.7%) |
| **SCS-sf** (1800) | 12 (2%) | 60 (10%) | 132 (22%) |
| **ELQ** (4950) | 6 (0.4%) | 171 (10.4%) | 233 (14.1%) |

EQ5D – EuroQol 5D-5L QOL measure; PSS – Perceived Stress Scale; MFIS – Modified Fatigue Impact Scale; MHI – Mental Health Inventory; PDQ – Perceived Deficits Questionnaire; MSSS – Modified Social Support Survey; PES – Pain Effects Scale; IVIS – Impact of Visual Impairment Scale; BCS – Bladder Control Scale; BWCS – Bowel Control Scale; MAAS – Mindful Attention Awareness Scale; SCSsf – Self-Compassion Scale – short form; ELQ – Emotional Lability Questionnaire.
