# Supplementary material for: Mindfulness-based stress reduction for people with multiple sclerosis – a feasibility randomised controlled trial
Source: BMC Neurol. 2017 May 16;17:94. doi: 10.1186/s12883-017-0880-8 (PMC5434553; doi:10.1186/s12883-017-0880-8)
Supplement: Supplementary file 5 — – Unadjusted RCT patient report outcome models. Tables S5.1–S5.5. provide detailed statistical data for unadjusted analyses. Figure S5.1 provides a forest plot for unadjusted treatment effects immediately post-MBSR, whilst Figure S5.2. provides a forest plot of unadjusted treatment effects three months post-MBSR. (DOCX 30 kb) [file 12883_2017_880_MOESM5_ESM.docx]

**Additional file 5 - Unadjusted RCT patient report outcome models**

Table S5.1: Unadjusted scores for primary outcome measures

|  | | Mean (SD) | | Change from baseline | | | Treatment effect* (95% CI), Significance  (*Intervention-control) | Effect size (Cohen’s ‘d’); 95% CI |
| --- | --- | --- | --- | --- | --- | --- | --- | --- |
| Measure | Time point | Intervention | Control | | Intervention | Control | N/A | N/A |
| EQ-5D (utility score) | Baseline | 0.53 (0.23) | 0.56 (0.27) | | N/A | N/A | N/A | N/A |
|  | Post | 0.55 (0.23) | 0.59 (0.23) | | 0.02 (0.18) | 0.05 (0.17) | -0.04 (-0.13 – 0.06), p=0.44 | -0.17 (-0.57 – 0.26) |
|  | F/u | 0.54 (0.24) | 0.58 (0.28) | | 0.01 (0.20) | 0.02 (0.17) | -0.02 (-0.13 – 0.09), p=0.67 | -0.08 (-0.50 – 0.35) |
| EQ5D - AUC | Baseline | N/A | N/A | | N/A | N/A | N/A | N/A |
|  | Post | 0.09 (0.04) | 0.10 (0.04) | | N/A | N/A | -0.00 (-0.03 – 0.02), p=0.82 | 0.00 (-0.13 – 0.09) |
|  | F/u | 0.24 (0.09) | 0.24 (0.10) | | N/A | N/A | -0.01 (-0.07 – 0.05), p=0.74 | 0.10 (-0.27 – 0.19) |

F/u – Follow up; N/A – Not applicable

Table S5.2: Unadjusted scores for primary outcome measures

|  | | Mean (SD) | | Change from baseline | | | Treatment effect* (95% CI), Significance  (*Intervention-control) | Effect size (Cohen’s ‘d’) (95% CI) |
| --- | --- | --- | --- | --- | --- | --- | --- | --- |
| Perceived stress scale - overall | Baseline | 21.08 (1.72) | 21.96 (1.34) | | N/A | N/A | N/A | N/A |
|  | Post | 13.50 (7.62) | 21.77 (8.01) | | -7.50 (-8.00) | -0.32 (-6.27) | -7.34 (-11.44 - -3.23), **p=<0.01** | 0.93 (0.41 – 1.44) |
|  | F/u | 16.05 (7.94) | 18.83 (5.93) | | -4.40 (7.16) | -2.87 (4.60) | -1.51 (-5.04 – 2.20), p=0.39 | 0.26 (-0.37 – 0.85) |
| Perceived stress scale – negative stressors | Baseline | 14.56 (6.09) | 14.60 (4.97) | | N/A | N/A | N/A | N/A |
|  | Post | 9.10 (5.26) | 14.23 (5.80) | | -5.00 (5.96) | -0.27 (4.76) | -4.92 (-7.86 - -1.98), **p<0.05** | 0.85 (0.34 – 1.35) |
|  | F/u | 10.15 (6.55) | 11.48 (5.30) | | -3.75 (5.81) | -3.13 (3.60) | -0.84 (-3.60 – 1.92), p=0.54 | 0.14 (-0.33 – 0.62) |
| Perceived stress scale – stress resilience | Baseline | 6.79 (2.82) | 7.36 (2.72) | | N/A | N/A | N/A | N/A |
|  | Post | 4.40 (2.58) | 7.54 (2.91) | | 2.50 (2.48) | 0.04 (2.64) | -2.75 (-4.21 - -1.30), **p<0.001** | 0.98 (0.46 – 1.49) |
|  | F/u | 5.90 (1.92) | 7.34 (1.72) | | 0.65 (2.28) | 0.26 (2.78) | -1.31 (-2.73 - -0.25), **p<0.05** | 0.51 (0.10 – 1.06) |

F/u – Follow up; N/A – Not applicable

Table S5.3: Unadjusted scores for Multiple Sclerosis Quality of Life Inventory

|  | | Mean (SD) | | Change from baseline | | Treatment effect* (95% CI), Significance  (*Intervention-control) | Effect size (Cohen’s ‘d’); 95% CI |
| --- | --- | --- | --- | --- | --- | --- | --- |
| Measure |  | Intervention | Control | Intervention | Control |  |  |
| MFIS | Baseline | 53.21 (18.68) | 54.26 (16.77) | N/A | N/A | N/A | N/A |
|  | Post | 38.47 (19.84) | 49.91 (17.19) | -13.33 (14.56) | -4.18 (10.87) | -9.46 (-17.24 - -1.67), **p<0.05** | 0.71 (0.13 – 1.29) |
|  | F/u | 42.95 (18.58) | 50.00 (15.54) | -9.5 (14.58) | -3.91 (11.15) | -5.72 (-13.10 – 1.66), p=0.13 | 0.44 (-1.00 - -0.13) |
| MHI | Baseline | 68.92 (17.48) | 67.45 (16.87) | N/A | N/A | N/A | N/A |
|  | Post | 83.52 (14.18) | 74.22 (16.53) | 13.43 (13.65) | 6.10 (13.00) | 8.38 (1.09 - 15.68), **p<0.01** | 0.61 (0.08 – 1.15) |
|  | F/u | 78.29 (19.60) | 73.41 (17.08) | 8.19 (18.44) | 6.25 (8.73) | 2.40 (-6.46 – 11.26), p=0.59 | 0.17 (-0.44 – 1.09) |
| PDQ | Baseline | 38.48 (16.24) | 39.04 (14.36) | N/A | N/A | N/A | N/A |
|  | Post | 29.05 (12.31) | 34.32 (14.81) | -9.00 (12.98) | -4.10 (8.77) | -5.02 (-11.27 – 1.23), p=0.11 | 0.46 (0.11- 1.03) |
|  | F/u | 33.18 (16.94) | 35.23 (14.65) | -5.47 (7.16) | -2.29 (7.71) | -2.93 (-8.00 – 2.12), p=0.25 | 0.38 (0.28 – 1.05) |
| MSSS | Baseline | 44.61 (25.05) | 43.12 (20.74) | N/A | N/A | N/A | N/A |
|  | Post | 45.60 (26.93) | 41.45 (17.01) | -3.83 (10.62) | -0.18 (15.69) | -1.90 (-10.08 – 6.28), p=0.64 | 0.14 (-0.74 – 0.46) |
|  | F/u | 44.00 (25.40) | 45.27 (21.36) | -6.56 (8.99) | 2.45 (16.98) | -7.76 (-16.28 – 0.76), p=0.07 | 0.53 (-1.12 – 0.05) |
| PES | Baseline | 17.76 (5.75) | 18.17 (6.04) | N/A | N/A | N/A | N/A |
|  | Post | 14.29 (4.71) | 17.00 (5.65) | -2.90 (4.13) | -1.18 (5.48) | -2.09 (-4.62 – 0.45), p=0.11 | -0.43 (-0.94 – 0.09) |
|  | F/u | 14.48 (5.73) | 17.22 (5.69) | -2.71 (4.82) | -1.00 (4.70) | -1.96 (-4.60 – 0.67), p=0.14 | -0.41 (-0.96 – 0.14) |
| IVIS | Baseline | 7.08 (3.29) | 7.54 (3.64) | N/A | N/A | N/A | N/A |
|  | Post | 6.55 (2.52) | 7.37 (3.12) | -0.85 (3.36) | -0.22 (3.16) | -0.78 (-2.32 – 0.76), p=0.31 | -0.24 (-0.72 – 0.24) |
|  | F/u | 6.90 (2.77) | 7.47 (3.30) | -0.38 (3.14) | -0.41 (3.23) | -0.14 (-1.67 – 1.38), p=0.85 | 0.04 (-0.53 – 0.44) |
| BCS | Baseline | 9.71 (6.15) | 9.33 (5.82) | N/A | N/A | N/A | N/A |
|  | Post | 7.86 (4.79) | 7.61 (4.67) | -1.75 (3.94) | -1.95 (3.72) | 0.13 (-1.76 – 2.02), p=0.89 | 0.03 (-0.47 – 0.53) |
|  | F/u | 7.90 (4.75) | 8.13 (5.21) | -1.32 (4.50) | -1.09 (3.15) | -0.33 (-2.37 – 1.71), p=0.74 | 0.09 (-0.63 – 0.45) |
| BWCS | Baseline | 11.87 (6.19) | 9.04 (6.43) | N/A | N/A | N/A | N/A |
|  | Post | 9.80 (5.25) | 8.09 (5.15) | -2.11 (2.97) | -1.09 (6.08) | 0.21 (-2.41 – 2.82), p=0.87 | 0.04 (-0.49 – 0.57) |
|  | F/u | 10.05 (5.58) | 8.09 (4.44) | -1.06 (5.24) | -1.22 (4.96) | 1.27 (-1.36 – 3.90), p=0.34 | 0.25 (-0.27 – 0.77) |
| SSS | Baseline | 13.00 (6.50) | 13.84 (6.87) | N/A | N/A | N/A | N/A |
|  | Post | 8.80 (3.88) | 14.89 (6.50) | -1.11 (4.34) | 0.23 (3.56) | -2.38 (-5.53 - -0.03), p=0.13 | 0.62 (0.01 -1.45) |
|  | F/u | 8.90 (4.43) | 14.69 (7.00) | -1.67 (2.50) | 1.64 (4.85) | -3.80 (-7.24 – - 0.01), **p<0.05** | 0.88 (0.01 – 1.67) |

F/u – Follow up; N/A – Not applicable

Table S5.4: Unadjusted scores for measures of Mindfulness and Self-Compassion

|  | | Mean (SD) | | Change from baseline | | | Treatment effect* (95% CI), Significance  (*Intervention-control) | Effect size (Cohen’s ‘d’); 95% CI |
| --- | --- | --- | --- | --- | --- | --- | --- | --- |
| Measure |  | Intervention | Control | | Intervention | Control |  |  |
| MAAS | Baseline | 52.09 (17.29) | 49.81 (10.89) | | N/A | N/A | N/A | N/A |
|  | Post | 57.76 (9.81) | 58.17 (14.06) | | 12.80 (10.14) | 8.5 (11.60) | 6.22 (-0.90 – 13.35), p=0.09 | 0.56 (-0.08 - 1.21) |
|  | F/u | 66.24 (7.64) | 51.70 (12.60 | | 15.6 (11.75) | 1.65 (7.52) | 14.67 (8.87 – 20.48), **p<0.001** | 1.23 (0.80 – 1.72) |
| SCS-sf | Baseline | 31.58 (10.17) | 32.56 (8.70) | | N/A | N/A | N/A | N/A |
|  | Post | 42.71 (9.80) | 33.79 (10.84) | | 11.00 (10.21) | 1.38 (8.21) | 9.44 (4.21 – 14.67), **p<0.01** | 0.92 (0.41 – 1.43) |
|  | F/u | 41.27 (11.75) | 32.15 (9.65) | | 9.18 (11.05) | 0.35 (7.34) | 8.99 (3.09 – 14.89) **p<0.01** | 0.89 (0.31 – 1.47) |

F/u – Follow up; N/A – Not applicable

Table S5.5: Unadjusted scores for the Emotional Lability Questionnaire

|  | | Mean (SD) | | Change from baseline | | Treatment effect* (95% CI), Significance  (*Intervention-control) | Effect size |
| --- | --- | --- | --- | --- | --- | --- | --- |
| Measure |  | Intervention | Control | Intervention | Control |  |  |
| ELQ | Baseline | 15.19 (14.18) | 15.46 (11.33) | N/A | N/A | N/A | N/A |
|  | Post | 9.56 (10.15) | 9.14 (9.56) | -5.79 (9.67) | -5.05 (8.21) | -0.55 (-5.88 – 4.76), p=0.83 | 0.06 (-0.46 – 0.37) |
|  | F/u | 8.72 (11.31) | 11.00 (8.29) | -5.25 (13.58) | -5.12 (8.01) | -1.22 (-7.03 – 4.60), p=0.67 | 0.12 (-0.41 – 0.27) |

F/u – Follow up; N/A – Not applicable

Figure S5.1: Unadjusted treatment effects with confidence intervals immediately post- MBSR**:**

EQ5D – EuroQol QOL measure; AUC – EuroQol Area Under the Curve; PSS – Perceived Stress Scale; MFIS – Modified Fatigue Impact Scale; MHI – Mental Health Inventory; PDQ – Perceived Deficits Questionnaire; MSSS – Modified Social Support Survey; PES – Pain Effects Scale; IVIS – Impact of Visual Impairment Scale; BCS – Bladder Control Scale; BWCS – Bowel Control Scale; SSS – Sexual Satisfaction Scale; MAAS – Mindful Attention Awareness Scale; SCS-sf – Self-Compassion Scale – short form; ELQ – Emotional Lability Questionnaire

Figure S5.2: Unadjusted treatment effects with confidence intervals three moths post-MBSR**:**

EQ5D – EuroQol QOL measure; AUC – EuroQol Area Under the Curve; PSS – Perceived Stress Scale; MFIS – Modified Fatigue Impact Scale; MHI – Mental Health Inventory; PDQ – Perceived Deficits Questionnaire; MSSS – Modified Social Support Survey; PES – Pain Effects Scale; IVIS – Impact of Visual Impairment Scale; BCS – Bladder Control Scale; BWCS – Bowel Control Scale; SSS – Sexual Satisfaction Scale; MAAS – Mindful Attention Awareness Scale; SCS-sf – Self-Compassion Scale – short form; ELQ – Emotional Lability Questionnaire
