# Supplementary material for: Mindfulness-based stress reduction for people with multiple sclerosis – a feasibility randomised controlled trial
Source: BMC Neurol. 2017 May 16;17:94. doi: 10.1186/s12883-017-0880-8 (PMC5434553; doi:10.1186/s12883-017-0880-8)
Supplement: Supplementary file 6 — - Adjusted RCT patient report outcome models. Tables S6.1–S6.9. provide detailed statistical data for adjusted analyses (age, sex, SES, previous meditation/yoga experience). (DOCX 41 kb) [file 12883_2017_880_MOESM6_ESM.docx]

**Additional file 6 - Tables S6.1-S6.9 with full details of statistics for each outcome measure**

**Table S6.1**: **Adjusted scores (Age, sex, deprivation, previous meditation/yoga experience) for perceived stress**

|  | | Mean (SD) | | Change from baseline | | | Treatment effect* (95% CI), Significance  (*Intervention-control) | Effect size (Cohen’s ‘d’) (95% CI) |
| --- | --- | --- | --- | --- | --- | --- | --- | --- |
| Measure | Time | Intervention | Control | | Intervention | Control |  |  |
| Perceived stress scale - overall | Baseline | 21.08 (1.72) | 21.96 (1.34) | | N/A | N/A | N/A | N/A |
|  | Post | 13.50 (7.62) | 21.77 (8.01) | | -7.50 (-8.00) | -0.32 (-6.27) | -7.34 (-11.44 - -3.23), **p=<0.01** | 0.93 (0.41 – 1.44) |
|  | F/u | 16.05 (7.94) | 18.83 (5.93) | | -4.40 (7.16) | -2.87 (4.60) | -1.51 (-5.04 – 2.20), p=0.39 | 0.26 (-0.37 – 0.85) |
| Perceived stress scale – negative stressors | Baseline | 14.56 (6.09) | 14.60 (4.97) | | N/A | N/A | N/A | N/A |
|  | Post | 9.10 (5.26) | 14.23 (5.80) | | -5.00 (5.96) | -0.27 (4.76) | -4.75 (-7.81 - -1.69), **p<0.05** | 0.82 (0.29 – 1.34) |
|  | F/u | 10.15 (6.55) | 11.48 (5.30) | | -3.75 (5.81) | -3.13 (3.60) | -0.56 (-3.61 – 2.49), p=0.71 | 0.12 (-0.52 - 0.77) |
| Perceived stress scale – stress resilience | Baseline | 6.79 (2.82) | 7.36 (2.72) | | N/A | N/A | N/A | N/A |
|  | Post | 4.40 (2.58) | 7.54 (2.91) | | -2.50 (2.48) | 0.04 (2.64) | -2.60 (-4.08 - -1.12), **p<0.05** | 0.92 (0.40 – 1.45) |
|  | F/u | 5.90 (1.92) | 7.34 (1.72) | | -0.65 (2.28) | 0.26 (2.78) | -1.17 (-2.34 - -0.02), p=0.05 | 0.46 (-0.01 – 0.91) |

F/u – Follow up; N/A – Not applicable

**Table S6.2**: **Adjusted scores (Age, sex, deprivation, previous meditation/yoga experience) for Quality of Life**

|  | | Mean (SD) | | Change from baseline | | | Treatment effect* (95% CI), Significance  (*Intervention-control) | Effect size (Cohen’s ‘d’) (95% CI) |
| --- | --- | --- | --- | --- | --- | --- | --- | --- |
| Measure | Time | Intervention | Control | | Intervention | Control |  |  |
| EQ-5D un-weighted | Baseline | 12.20 (3.21) | 11.36 (4.20) | | N/A | N/A | N/A | N/A |
|  | Post | 11.62 (3.22) | 11.43 (3.88) | | -0.71 (2.00) | -0.13 (2.94) | -0.34 (-1.88 – 1.21), p=0.66 | 0.13 (-0.48 – 0.74) |
|  | F/u | 11.62 (3.57) | 11.35 (4.41) | | -0.71 (2.01) | 0.04 (2.72) | -0.57 (-2.17 – 1.04), p=0.48 | 0.23 (-0.43 – 0.89) |
| EQ-5D weighted | Baseline | 0.53 (0.23) | 0.56 (0.27) | | N/A | N/A | N/A | N/A |
|  | Post | 0.55 (0.23) | 0.59 (0.23) | | 0.02 (0.18) | 0.05 (0.17) | -0.04 (-0.14 – 0.07), p=0.48 | 0.17 (-0.3 – 0.61) |
|  | F/u | 0.54 (0.24) | 0.58 (0.28) | | 0.01 (0.20) | 0.02 (0.17) | -0.02 (-0.13 – 0.09), p=0.71 | 0.08 (-0.35 – 0.50) |
| EQ-5D – Area under the curve | Baseline | N/A | N/A | | N/A | N/A | N/A | N/A |
|  | Post | 0.09 (0.04) | 0.10 (0.04) | | N/A | N/A | 0.00 (-0.03 – 0.02), p=0.80 | 0.00 (-0.50 – 0.75) |
|  | F/u | 0.24 (0.09) | 0.24 (0.10) | | N/A | N/A | -0.01 (-0.08 – 0.05), p=0.71 | 0.10 (-0.50 – 0.80) |
| EQ-5D - Mobility | Baseline | 2.68 (1.03) | 2.44 (1.16) | | N/A | N/A | N/A | N/A |
|  | Post | 2.67 (1.24) | 2.48 (1.12) | | -0.14 (0.58) | 0.00 (0.85) | -0.05 (-0.53 – 0.44), p=0.85 | 0.07 (-0.60 – 0.73) |
|  | F/u | 2.81 (1.08) | 2.43 (1.12) | | 0.00 (0.55) | 0.04 (0.64) | 0.07 (-0.31 – 0.46), p=0.70 | 0.11 (-0.78 – 0.53) |
| EQ-5D – Self-care | Baseline | 1.72 (0.89) | 1.64 (0.95) | | N/A | N/A | N/A | N/A |
|  | Post | 1.81 (0.81) | 1.70 (0.93) | | 0.00 (0.71) | 0.00 (0.60) | 0.02 (-0.38 – 0.42), p=0.92 | 0.03 (-0.65 – 0.58) |
|  | F/u | 1.76 (0.89) | 1.70 (0.88) | | 0.74 (-0.05) | 0.09 (0.73) | -0.07 (-0.51 – 0.38), p=0.77 | 0.10 (-0.52 – 0.70) |
| EQ-5D – Usual activities | Baseline | 2.64 (0.91) | 2.44 (1.19) | | N/A | N/A | N/A | N/A |
|  | Post | 2.57 (0.87) | 2.48 (1.12) | | 0.00 (0.89) | 0.00 (1.04) | 0.01 (-0.55 – 0.57), p=0.97 | 0.01 (-0.59 – 0.57) |
|  | F/u | 2.52 (1.08) | 2.57 (1.20) | | -0.05 (0.86) | 0.13 (0.97) | -0.11 (-0.67 – 0.46), p=0.69 | 0.12 (-0.51 – 0.74) |
| EQ-5D – Pain/ discomfort | Baseline | 2.72 (1.17) | 2.60 (1.15) | | N/A | N/A | N/A | N/A |
|  | Post | 2.62 (0.97) | 2.61 (1.03) | | -0.10 (0.44) | -0.04 (0.77) | 0.03 (-0.32 – 0.39), p=0.86 | 0.05 (-0.63 – 0.52) |
|  | F/u | 2.57 (1.03) | 2.52 (1.16) | | -0.14 (0.57) | -0.09 (0.73) | -0.04 (-0.45 – 0.37), p=0.85 | 0.06 (-0.57 – 0.69) |
| EQ-5D – Anxiety/ depression | Baseline | 2.44 (0.82) | 2.24 (0.83) | | N/A | N/A | N/A | N/A |
|  | Post | 1.95 (0.67) | 2.17 (0.89) | | -0.47 (0.68) | -0.09 (0.73) | -0.30 (-0.71 – 0.12), p=0.16 | 0.41 (-0.16 – 0.97) |
|  | F/u | 1.95 (0.97) | 2.13 (0.97) | | -0.48 (0.93) | -0.13 (0.69) | -0.21 (-0.74 – 0.31), p=0.42 | 0.26 (-0.38 – 0.90) |

F/u – Follow up; N/A – Not applicable

**Table S6.3**: **Adjusted scores (Age, sex, deprivation, previous meditation/yoga experience) for overall MSQLI scores**

|  | | Mean (SD) | | Change from baseline | | Treatment effect* (95% CI), Significance  (*Intervention-control) | Effect size (Cohen’s ‘d’) (95% CI) |
| --- | --- | --- | --- | --- | --- | --- | --- |
| Measure | Time | Intervention | Control | Intervention | Control |  |  |
| Modified fatigue impact scale - MFIS | Baseline | 53.21 (18.68) | 54.26 (16.77) | N/A | N/A | N/A | N/A |
|  | Post | 38.47 (19.84) | 49.91 (17.19) | -13.33 (14.56) | -4.18 (10.87) | -8.32 (-16.87 – 0.24), p=0.06 | 0.62 (-0.02 – 1.12) |
|  | F/u | 42.95 (18.58) | 50.00 (15.54) | -9.5 (14.58) | -3.91 (11.15) | -4.24 (-12.21 – 3.75), p=0.29 | 0.33 (-0.29 – 0.94) |
| Mental health inventory - MHI | Baseline | 68.92 (17.48) | 67.45 (16.87) | N/A | N/A | N/A | N/A |
|  | Post | 83.52 (14.18) | 74.22 (16.53) | 13.43 (13.65) | 6.10 (13.00) | 7.34 (-0.36 – 15.05), p=0.06 | 0.54 (-0.03 – 1.10) |
|  | F/u | 78.29 (19.60) | 73.41 (17.08) | 8.19 (18.44) | 6.25 (8.73) | 2.08 (-7.46 – 11.62), p=0.66 | 0.14 (- 0.52 – 0.81) |
| Perceived deficits scale - PDQ | Baseline | 38.48 (16.24) | 39.04 (14.36) | N/A | N/A | N/A | N/A |
|  | Post | 29.05 (12.31) | 34.32 (14.81) | -9.00 (12.98) | -4.10 (8.77) | -4.77 (-11.17 – 1.64), p=0.14 | 0.43 (-0.15 – 1.02) |
|  | F/u | 33.18 (16.94) | 35.23 (14.65) | -5.47 (7.16) | -2.29 (7.71) | -2.21 (-7.54 – 3.13), p=0.41 | 0.29 (-0.41 – 1.00) |
| Modified social support survey - MSSS | Baseline | 44.61 (25.05) | 43.12 (20.74) | N/A | N/A | N/A | N/A |
|  | Post | 45.60 (26.93) | 41.45 (17.01) | 0.99 (10.62) | -1.67 (15.69) | 0.47 (-8.05 – 8.99), p=0.91 | 0.03 (-0.59 – 0.66) |
|  | F/u | 44.00 (25.40) | 45.27 (21.36) | 0.61 (8.99) | 2.15 (16.98) | -5.60 (-14.34 – 3.15), p=0.20 | -0.39 (-0.99 – 0.22) |
| Pain effects scale - PES | Baseline | 17.76 (5.75) | 18.17 (6.04) | N/A | N/A | N/A | N/A |
|  | Post | 14.29 (4.71) | 17.00 (5.65) | -3.47 (4.13) | -1.17 (5.48) | -1.90 (-4.41 – 0.61), p=0.13 | 0.44 (-0.14 – 1.01) |
|  | F/u | 14.48 (5.73) | 17.22 (5.69) | -3.28 (4.82) | -0.95 (4.70) | -1.56 (-4.35 – 1.23), p=0.27 | 0.32 (-0.25 – 0.88) |
| Impact of visual impairment scale - IVIS | Baseline | 7.08 (3.29) | 7.54 (3.64) | N/A | N/A | N/A | N/A |
|  | Post | 6.55 (2.52) | 7.37 (3.12) | -0.53 (3.36) | -0.17 (3.16) | -0.80 (-2.21 – 0.61), p=0.26 | 0.30 (-0.23 – 0.82) |
|  | F/u | 6.90 (2.77) | 7.47 (3.30) | -0.18 (3.14) | -0.07 (3.23) | 0.11 (-1.46 – 1.25), p=0.91 | 0.04 (-0.48 – 0.56) |
| Bladder control scale - BCS | Baseline | 9.71 (6.15) | 9.33 (5.82) | N/A | N/A | N/A | N/A |
|  | Post | 7.86 (4.79) | 7.61 (4.67) | -1.85 (3.94) | -1.72 (3.72) | 0.51 (-1.56 – 2.58), p=0.62 | 0.13 (-0.41 – 0.68) |
|  | F/u | 7.90 (4.75) | 8.13 (5.21) | -1.81 (4.50) | -1.20 (3.15) | -0.20 (-2.33 – 1.90), p=0.85 | 0.05 (-0.50 – 0.62) |
| Bowel control scale - BWCS | Baseline | 11.87 (6.19) | 9.04 (6.43) | N/A | N/A | N/A | N/A |
|  | Post | 9.80 (5.25) | 8.09 (5.15) | -2.07 (2.97) | -0.95 (6.08) | 0.77 (-2.02 – 3.56), p=0.58 | 0.16 (-0.41 – 0.72) |
|  | F/u | 10.05 (5.58) | 8.09 (4.44) | -1.82 (5.24) | -0.95 (4.96) | 1.40 (-1.37 – 4.17), p=0.31 | 0.28 (-0.27 – 0.83) |
| Sexual satisfaction scale - SSS | Baseline | 13.00 (6.50) | 13.84 (6.87) | N/A | N/A | N/A | N/A |
|  | Post | 8.80 (3.88) | 14.89 (6.50) | 4.20 (4.34) | -1.05 (3.56) | -2.68 (-6.11 – 0.75), p=0.12 | 0.70 (-0.20 – 1.60) |
|  | F/u | 8.90 (4.43) | 14.69 (7.00) | -4.10 (2.50) | -0.85 (4.85) | -2.46 (-6.01 – 1.08), p=0.16 | 0.57 (-0.25 – 1.38) |

**Table S6.4**: **Adjusted scores (Age, sex, deprivation, previous meditation/yoga experience) for fatigue**

|  | | Mean (SD) | | Change from baseline | | Treatment effect* (95% CI), Significance  (*Intervention-control) | Effect size (Cohen’s ‘d’) (95% CI) |
| --- | --- | --- | --- | --- | --- | --- | --- |
| Measure | Time | Intervention | Control | Intervention | Control |  |  |
| Modified fatigue impact scale - overall | Baseline | 53.21 (18.68) | 54.26 (16.77) | N/A | N/A | N/A | N/A |
|  | Post | 38.47 (19.84) | 49.91 (17.19) | -13.33 (14.56) | -4.18 (10.87) | -8.32 (-16.87 – 0.24), p=0.06 | 0.62 (-0.02 – 1.12) |
|  | F/u | 42.95 (18.58) | 50.00 (15.54) | -9.5 (14.58) | -3.91 (11.15) | -4.24 (-12.21 – 3.75), p=0.29 | 0.33 (-0.29 – 0.94) |
| Modified fatigue impact scale - cognitive | Baseline | 24.04 (8.89) | 23.75 (7.18) | N/A | N/A | N/A | N/A |
|  | Post | 16.90 (8.49) | 22.04 (8.55) | -6.55 (7.03) | -1.77 (5.87) | -3.91 (-8.09 – 0.27), p=0.07 | 0.57 (-0.04 - 1.19) |
|  | F/u | 17.80 (9.51) | 21.17 (7.37) | -5.25 (7.13) | -2.36 (5.54) | -1.73 (-5.67 – 2.19), p=0.38 | 0.27 (-0.34 - 0.88) |
| Modified fatigue impact scale - physical | Baseline | 24.68 (9.30) | 26.92 (11.22) | N/A | N/A | N/A | N/A |
|  | Post | 18.32 (10.08) | 23.96 (8.23) | -6.16 (6.94) | -3.00 (7.64) | -3.36 (-7.36 – 0.62), p=0.10 | 0.45 (-0.08 – 0.99) |
|  | F/u | 21.19 (8.05) | 24.13 (8.08) | -3.48 (7.53) | -2.56 (8.41) | -1.38 (-5.35 – 2.60), p=0.49 | 0.17 (-0.33 – 0.67) |
| Modified fatigue impact scale - psychosocial | Baseline | 4.72 (2.42) | 5.08 (2.30) | N/A | N/A | N/A | N/A |
|  | Post | 3.45 (2.30) | 4.54 (2.04) | -1.40 (1.79) | -0.54 (1.47) | -0.82 (-1.77 – 0.12), p=0.09 | 0.49 (-0.07 - 1.07) |
|  | F/u | 3.95 (2.40) | 4.70 (2.01) | -0.90 (1.97) | -0.39 (1.47) | -0.50 (-1.51 – 0.51), p=0.32 | 0.23 (-0.30 – 0.87) |

F/u – Follow up; N/A – Not applicable

**Table S6.5**: **Adjusted scores (Age, sex, deprivation, previous meditation/yoga experience) for mental health**

|  | | Mean (SD) | | Change from baseline | | Treatment effect* (95% CI), Significance  (*Intervention-control) | Effect size (Cohen’s ‘d’) (95% CI) |
| --- | --- | --- | --- | --- | --- | --- | --- |
| Measure | Time | Intervention | Control | Intervention | Control |  |  |
| Mental health inventory - overall | Baseline | 68.92 (17.48) | 67.45 (16.87) | N/A | N/A | N/A | N/A |
|  | Post | 83.52 (14.18) | 74.22 (16.53) | 13.43 (13.65) | 6.10 (13.00) | 7.34 (-0.36 – 15.05), p=0.06 | 0.54 (-0.03 – 1.10) |
|  | F/u | 78.29 (19.60) | 73.41 (17.08) | 8.19 (18.44) | 6.25 (8.73) | 2.08 (-7.46 – 11.62), p=0.66 | 0.14 (- 0.52 – 0.81) |
| Mental health inventory - anxiety | Baseline | 51.84 (23.79) | 51.84 (17.45) | N/A | N/A | N/A | N/A |
|  | Post | 72.76 (17.23) | 59.65 (24.77) | 18.86 (20.20) | 8.17 (17.87) | 11.15 (-0.04 – 22.34), p=0.05 | 0.85 (-0.01 – 1.69) |
|  | F/u | 67.43 (23.91) | 57.56 (22.84) | 13.52 (21.45) | 6.26 (13.20) | 7.96 (-2.88 – 18.81), p=0.15 | 0.82 (-0.29 – 1.93) |
| Mental health inventory - depression | Baseline | 64.60 (23.14) | 60.42 (24.04) | N/A | N/A | N/A | N/A |
|  | Post | 78.57 (17.19) | 64.38 (20.18) | 11.90 (17.43) | 3.91 (19.24) | 10.41 (0.84 – 20.00), **p<0.05** | 1.35 (0.11 – 2.59) |
|  | F/u | 70.71 (23.04) | 65.68 (22.43) | 4.04 (21.72) | 5.00 (14.83) | -0.03 (-11.75 – 11.70), p=1.00 | 0.01 (-2.60 – 2.59) |
| Mental health inventory – behavior control | Baseline | 62.80 (25.17) | 57.29 (21.97) | N/A | N/A | N/A | N/A |
|  | Post | 78.33 (17.56) | 68.33 (22.44) | 14.29 (21.29) | 9.57 (14.30) | 6.01 (-3.80 – 15.81), p=0.22 | 0.51(-0.32 – 1.33) |
|  | F/u | 74.29 (23.84) | 71.08 (20.34) | 10.24 (24.92) | 11.81 (13.68) | -1.73 (-13.85 – 10.39), p=0.77 | 0.16 (-1.25 – 0.94) |
| Mental health inventory – positive affect | Baseline | 46.60 (21.10) | 48.00 (23.23) | N/A | N/A | N/A | N/A |
|  | Post | 62.62 (17.86) | 53.12 (20.68) | 14.29 (18.05) | 4.37 (20.13) | 7.80 (-2.39 – 18.00), p=0.13 | 0.87 (-0.27 – 2.00) |
|  | F/u | 55.24 (25.37) | 51.52 (21.71) | 6.90 (25.22) | 1.96 (16.57) | 3.91 (-8.92 – 16.75), p=0.54 | 0.90 (-1.88 – 3.89) |

F/u – Follow up; N/A – Not applicable

**Table S6.6: Adjusted scores (Age, sex, deprivation, previous meditation/yoga experience) for cognitive function**

|  | | Mean (SD) | | Change from baseline | | Treatment effect* (95% CI), Significance  (*Intervention-control) | Effect size (Cohen’s ‘d’) (95% CI) |
| --- | --- | --- | --- | --- | --- | --- | --- |
| Measure | Time | Intervention | Control | Intervention | Control |  |  |
| Perceived deficits questionnaire - overall | Baseline | 38.48 (16.24) | 39.04 (14.36) | N/A | N/A | N/A | N/A |
|  | Post | 29.05 (12.31) | 34.32 (14.81) | -9.00 (12.98) | -4.10 (8.77) | -4.77 (-11.17 – 1.64), p=0.14 | 0.43 (-0.15 – 1.02) |
|  | F/u | 33.18 (16.94) | 35.23 (14.65) | -5.47 (7.16) | -2.29 (7.71) | -2.21 (-7.54 – 3.13), p=0.41 | 0.29 (-0.41 – 1.00) |
| Perceived deficits questionnaire - attention | Baseline | 11.74 (4.18) | 11.08 (3.82) | N/A | N/A | N/A | N/A |
|  | Post | 8.75 (3.08) | 10.22 (4.25) | -2.72 (2.89) | -1.05 (2.05) | -1.59 (-3.05 - -0.12), **p<0.05** | 0.62 (0.05 – 1.19) |
|  | F/u | 9.00 (4.61) | 9.91 (4.05) | -2.11 (2.72) | -1.05 (2.46) | -0.70 (-2.37 – 0.97), p=0.40 | 0.23 (-0.37 – 0.91) |
| Perceived deficits questionnaire - retrospective memory | Baseline | 9.16 (4.5) | 9.16 (4.10) | N/A | N/A | N/A | N/A |
|  | Post | 7.30 (3.66) | 8.67 (4.12) | -1.65 (3.78) | -0.58 (2.84) | -1.24 (-3.02 – 0.53), p=0.17 | 0.37 (-0.16 – 0.91) |
|  | F/u | 7.57 (4.56) | 8.68 (4.02) | -1.33 (2.99) | -0.18 (2.63) | -0.99 (-2.81 – 0.83), p=0.28 | 0.35 (-0.29 – 0.99) |
| Perceived deficits questionnaire – prospective memory | Baseline | 7.42 (3.81) | 7.28 (3.59) | N/A | N/A | N/A | N/A |
|  | Post | 5.75 (2.90) | 7.25 (3.77) | -1.58 (3.29) | -0.05 (2.33) | -1.61 (-3.25 – 0.03), p=0.05 | 0.56 (-0.01 – 1.14) |
|  | F/u | 6.00 (4.02) | 7.05 (4.05) | -2.00 (2.40) | -0.09 (1.87) | -1.88 (-3.34 - -0.41), **p<0.05** | 0.81 (0.18 – 1.45) |
| Perceived deficits questionnaire – planning/ organisation | Baseline | 9.80 (5.25) | 10.48 (3.75) | N/A | N/A | N/A | N/A |
|  | Post | 7.15 (3.99) | 8.87 (3.67) | -2.15 (4.23) | -1.57 (3.64) | -1.15 (-3.21 – 0.92), p=0.27 | 0.29 (-0.24 – 0.85) |
|  | F/u | 7.90 (4.89) | 9.59 (3.80) | -1.70 (3.18) | -0.64 (2.52) | -0.90 (-2.64 – 0.84), p=0.30 | 0.31 (-0.29 – 0.92) |

F/u – Follow up; N/A – Not applicable

**Table S6.7: Adjusted scores (Age, sex, deprivation, previous meditation/yoga experience) for social support**

|  | | Mean (SD) | | Change from baseline | | Treatment effect* (95% CI), Significance  (*Intervention-control) | Effect size (Cohen’s ‘d’) (95% CI) |
| --- | --- | --- | --- | --- | --- | --- | --- |
| Measure | Time | Intervention | Control | Intervention | Control |  |  |
| Modified social support survey - overall | Baseline | 44.61 (25.05) | 43.12 (20.74) | N/A | N/A | N/A | N/A |
|  | Post | 45.60 (26.93) | 41.45 (17.01) | 0.99 (10.62) | -1.67 (15.69) | 0.47 (-8.05 – 8.99), p=0.91 | 0.03 (-0.59 – 0.66) |
|  | F/u | 44.00 (25.40) | 45.27 (21.36) | 0.61 (8.99) | 2.15 (16.98) | -5.60 (-14.34 – 3.15), p=0.20 | -0.39 (-0.99 – 0.22) |
| Modified social support survey - tangible | Baseline | 58.85 (35.69) | 57.50 (33.95) | N/A | N/A | N/A | N/A |
|  | Post | 64.88 (39.99) | 59.78 (32.36) | 4.68 (21.73) | 3.53 (20.11) | 5.30 (-7.86 – 18.46), p=0.42 | 0.26 (-0.38 – 0.89) |
|  | F/u | 62.20 (37.42) | 59.23 (35.55) | 0.63 (19.12) | 2.98 (26.77) | 1.07 (-13.51 – 15.56), p=0.88 | 0.05 (-0.58 – 0.67) |
| Modified social support survey - emotional | Baseline | 63.88 (34.07) | 62.13 (29.61) | N/A | N/A | N/A | N/A |
|  | Post | 64.38 (38.06) | 63.32 (29.25) | -4.84 (22.06) | 0.27 (27.06) | 0.29 (-15.53 – 16.00), p=0.98 | 0.01 (-0.63 – 0.64) |
|  | F/u | 60.27 (34.73) | 63.32 (28.90) | -6.84 (22.91) | 0.82 (23.50) | -5.12 (-19.27 – 9.04), p=0.47 | -0.22 (-0.83 – 0.39) |
| Modified social support survey - affection | Baseline | 53.67 (36.65) | 53.00 (33.50) | N/A | N/A | N/A | N/A |
|  | Post | 57.54 (41.07) | 53.82 (28.34) | 1.59 (28.21) | 24.08 (0.00) | 3.43 (-11.55 – 18.41), p=0.65 | 0.13 (-0.45 – 0.71) |
|  | F/u | 58.73 (38.23) | 60.98 (36.50) | 2.78 (32.10) | 7.19 (32.76) | -1.83 (-20.68 – 17.01), p=0.85 | 0.06 (-0.53 – 0.64) |
| Modified social support survey – positive interactions | Baseline | 69.10 (38.58) | 64.00 (32.52) | N/A | N/A | N/A | N/A |
|  | Post | 61.11 (35.19) | 55.80 (23.89) | -13.33 (20.30) | -6.16 (24.26) | 0.85 (-10.74 – 12.44), p=0.88 | 0.04 (-0.48 – 0.55) |
|  | F/u | 66.67 (37.46) | 64.13 (34.85) | -10.96 (19.26) | -0.36 (29.46) | -2.85 (-17.37 – 11.66), p=0.69 | 0.11 (-0.45 - 0.69) |

F/u – Follow up; N/A – Not applicable

**Table S6.8: Adjusted scores (Age, sex, deprivation, previous meditation/yoga experience) for measures of mindfulness and self-compassion**

|  | | Mean (SD) | | Change from baseline | | | Treatment effect* (95% CI), Significance  (*Intervention-control) | Effect size (Cohen’s ‘d’) (95% CI) |
| --- | --- | --- | --- | --- | --- | --- | --- | --- |
| Measure |  | Intervention | Control | | Intervention | Control |  |  |
| Mindful attention awareness scale - MAAS | Baseline | 52.09 (17.29) | 49.81 (10.89) | | N/A | N/A | N/A | N/A |
|  | Post | 57.76 (9.81) | 58.17 (14.06) | | 12.80 (10.14) | 8.5 (11.60) | 6.62 (-1.13 – 14.36), p=0.09 | 0.6 (-0.10 – 1.30) |
|  | F/u | 66.24 (7.64) | 51.70 (12.60 | | 15.6 (11.75) | 1.65 (7.52) | 14.45 (8.01 – 20.84), **p<0.001** | 1.21 (0.67 – 1.75) |
| Self-compassion scale – short form - SCS-sf | Baseline | 31.58 (10.17) | 32.56 (8.70) | | N/A | N/A | N/A | N/A |
|  | Post | 42.71 (9.80) | 33.79 (10.84) | | 11.00 (10.21) | 1.38 (8.21) | 7.97 (2.39-13.55), **p<0.01** | 0.78 (0.23 – 1.32) |
|  | F/u | 41.27 (11.75) | 32.15 (9.65) | | 9.18 (11.05) | 0.35 (7.34) | 8.15 (1.80 – 14.50) **p<0.05** | 0.80 (0.18 – 1.43) |

F/u – Follow up; N/A – Not applicable

**Table S6.9: Adjusted scores (Age, sex, deprivation, previous meditation/yoga experience) for emotional lability**

|  | | Mean (SD) | | Change from baseline | | Treatment effect* (95% CI), Significance  (*Intervention-control) | Effect size |
| --- | --- | --- | --- | --- | --- | --- | --- |
| Measure | Time | Intervention | Control | Intervention | Control |  |  |
| ELQ | Baseline | 15.19 (14.18) | 15.46 (11.33) | N/A | N/A | N/A | N/A |
|  | Post | 9.56 (10.15) | 9.14 (9.56) | -5.63 (9.67) | -6.32 (8.21) | -0.55 (-6.47 – 5.36), p=0.85 | 0.06 (-0.42 – 0.51) |
|  | F/u | 8.72 (11.31) | 11.00 (8.29) | -6.47 (13.58) | -4.46 (8.01) | -0.79 (-6.76 – 5.18), p=0.79 | 0.07 (-0.39 – 0.30) |

F/u – Follow up; N/A – Not applicable
